# Inhibitors of the Glyoxylate Cycle Enzyme ICL1 in *Candida albicans* for Potential Use as Antifungal Agents

**DOI:** 10.1371/journal.pone.0095951

**Published:** 2014-04-29

**Authors:** Hong-Leong Cheah, Vuanghao Lim, Doblin Sandai

**Affiliations:** 1 Infectomic Cluster, Advanced Medical & Dental Institute, Universiti Sains Malaysia, Penang, Malaysia; 2 Integrative Medicine Cluster, Advanced Medical and Dental Institute, Universiti Sains Malaysia, Penang, Malaysia; Institute of Microbiology, Switzerland

## Abstract

*Candida albicans* is an opportunistic pathogen that causes candidiasis in humans. In recent years, metabolic pathways in *C. albicans* have been explored as potential antifungal targets to treat candidiasis. The glyoxylate cycle, which enables *C. albicans* to survive in nutrient-limited host niches and its. Key enzymes (e.g., isocitrate lyase (ICL1), are particularly attractive antifungal targets for *C. albicans*. In this study, we used a new screening approach that better reflects the physiological environment that *C. albicans* cells experience during infection to identify potential inhibitors of ICL. Three compounds (caffeic acid (CAFF), rosmarinic acid (ROS), and apigenin (API)) were found to have antifungal activity against *C. albicans* when tested under glucose-depleted conditions. We further confirmed the inhibitory potential of these compounds against ICL using the ICL enzyme assay. Lastly, we assessed the bioavailability and toxicity of these compounds using Lipinski's rule-of-five and ADMET analysis.

## Introduction

*Candida albicans* and other medically relevant *Candida* species are mainly common commensal yeasts that inhabit mucosal surfaces and the gastrointestinal and genitourinary tracts [1]. They are usually benign but can become infectious if an environmental niche becomes available or the host immune system becomes impaired [2]. *C. albicans* causes two types of infections: superficial infections, such as oral thrush and vaginal candidiasis, and potentially fatal systemic candidiasis [3]. Candidiasis is among the most common nosocomial systemic infections, with mortality rates as high as 50% [1], [3], [4]. Several virulence attributes, including adhesins and invasins, polymorphism, phenotypic switching, extracellular hydrolytic enzymes, and biofilm formation, as well as fitness attributes such as metabolic flexibility, contribute to the pathogenicity of *C. albicans* [4], [5].

Choices of antifungal drugs to treat candidiasis are limited. Those that are in routine clinical use include the polyenes, azoles, and echinocandins [2]. Polyenes (e.g., amphotericin B) bind to ergosterol in the plasma membrane, azoles (e.g., fluconazole) inhibit ergosterol synthesis, and the echinocandins inhibit glucan synthesis [2], [6]. The search for novel antifungal drugs with no significant side effects on patients continues, and research has been focused mainly on specific cell components and pathways, such as cell wall and ergosterol synthesis. However, in recent years metabolic pathways have been explored as potential antifungal targets, as they contribute to the metabolic flexibility that allows cells to survive in nutrient-limited host niches during infection [4]. Metabolic flexibility is particularly crucial for *C. albicans* and other pathogenic fungi for survival in nutrient-limited host niches as it contributes to effective assimilation of different carbon sources [7]. It was previously reported that metabolic flexibility of *C. albicans* not only contributes to adaptation and survival in host niches but also affects pathogenicity and virulence [4], [8]. Glycolysis, gluconeogenesis, and the glyoxylate cycle are all thought to contribute to survival of *C. albicans* during infection, but their specific mechanisms remain poorly understood.

**Figure 1:**
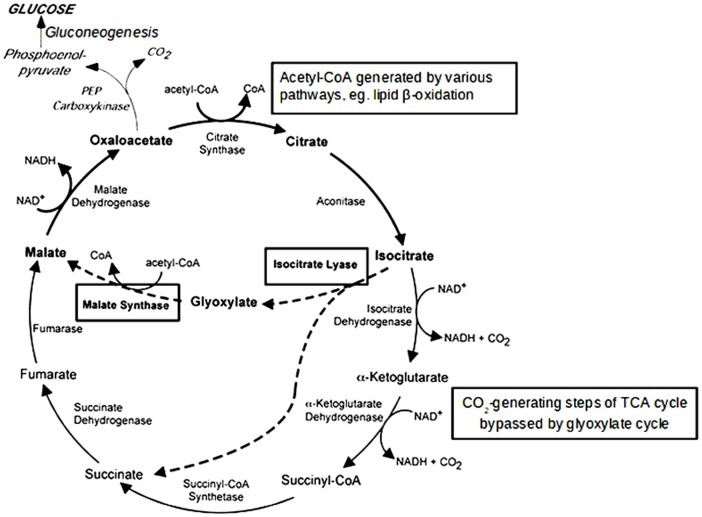
TCA cycle (black arrows) and glyoxylate cycle (dashed arrows). In both cycles, oxaloacetate serves as the precursor for gluconeogenesis, but the glyoxylate cycle bypasses the carbon dioxide generating steps of the TCA cycle via isocitrate lyase and malate synthase, thus conserving the carbons for gluconeogenesis. Adapted from Lorenz and Fink (2002) [9].

Among the metabolic pathways, the glyoxylate cycle has been studied the most. The glyoxylate cycle is a modified tricarboxylic acid (TCA) cycle that bypasses the CO_2_-generating steps to conserve carbons as substrates for gluconeogenesis, during which they are incorporated into new molecules of glucose [9], [10] (Figure 1). The key enzymes for this pathway, isocitrate lyase (ICL) and malate synthase, are highly conserved among bacteria, plants, fungi, and nematodes [10], [11]. In a glucose-depleted environment, the conservation of carbons is important for cell survival. The glyoxylate cycle enables *C. albicans* to survive and grow in the nutrient-limited environment inside phagocytic cells such as macrophages and neutrophils by utilizing alternative carbon sources such as lipids and amino acids [10], [12], [13]. It was previously reported that the glyoxylate cycle of *C. albicans* is activated when cells are engulfed by macrophages and neutrophils [14], [15], [16], [17]. In a study using ICL-deficient mutants in a mouse model, [14] found that this enzyme is essential for virulence.

Given that the glyoxylate cycle is essential for *C. albicans* to survive in host niches, key enzymes such as ICL are attractive potential drug targets. ICL has been explored as a potential drug target in other pathogenic fungi [18], *Mycobacterium tuberculosis* [19], [20], [21], and *Burkholderia* species [22], [23]. Importantly, no human ortholog of this pathway or its respective enzymes has been identified, which makes it a promising antifungal target to treat *C. albicans* infection. In this study, ICL of *C. albicans* was selected as the antifungal target for drug screening using a collection of selected plant reference compounds. Because ICL is essential when glucose is depleted, we sought to exploit this phenotype by screening the compounds for antifungal properties in a defined minimal medium (i.e., yeast nitrogen base (YNB) supplemented with lactate as the sole carbon source). Such an alternative screening approach can identify new compounds among existing compounds that have previously shown no antifungal property when screened in glucose-supplemented medium. We also studied the drug-likeness and potential toxicity effect of the potential ICL inhibitors using *in silico* analysis.

## Materials and Methods

### *Candida albicans* Strain and Media

*Candida albicans* ATCC10231 was obtained from laboratory culture stocks. The yeast was maintained in YPD (1% yeast extract, 2% peptone, 2% D-glucose) medium prior to use in the experiments. For the alternative screening approach, the yeast was cultivated in minimal defined medium (0.67% yeast nitrogen base) supplemented with 2% lactate or 2% D-glucose as the sole carbon source (abbreviated as YNBL and YNBG, respectively).

### Antifungal Drug and Reference Compounds

**Figure 2:**
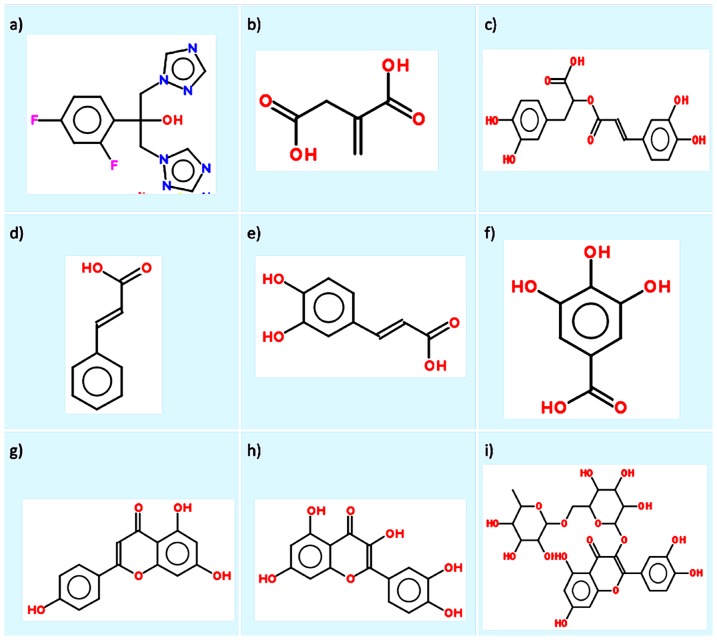
Drugs and plant reference compounds used in this study with their PubChem IDs.

The antifungal drug used as the control in this study, fluconazole (FLC), and the plant reference compounds, itaconic acid (ITC), quercetin (QCT), cinnamic acid (CINN), rutin (RT), caffeic acid (CAFF), gallic acid (GALL), apigenin (API), and rosmarinic acid (ROS) (Figure 2), were purchased from Sigma-Aldrich Co. (St. Louis, MO, USA). For the experiments, stock solution of FLC was prepared in sterile distilled water, whereas stock solutions of reference compounds were prepared in DMSO. All stock solutions were stored at −80°C until used.

### Alternative Screening Approach

The primary screening of the plant reference compounds at a final concentration of 1000 µg/mL was conducted in YNBL with *C. albicans* ATCC10231 in a 96-well plate format. For internal control purposes, each plate included wells with drug-free control, FLC treatment, and ITC (a known ICL inhibitor) treatment. The yeast was cultivated in each well at a final volume of 200 µL with final cell density of 0.5–2.5×10^5^ cfu/mL. The plates were incubated at 35°C for 24 hours before being measured spectrophotometrically at 530 nm on Sunrise™ microplate absorbance reader (Tecan Group Ltd., Männedorf, Switzerland). Growth in each compound was calculated in percentage relative to the drug-free control included in each plate. All reference compounds were then subjected to secondary screening in YNBL, which was conducted in triplicate. Growth assessment in YNBG was also conducted during this secondary screening phase of the experiment. Any compounds confirmed as potential ICL inhibitors were further characterized for specific ICL-inhibition as described below.

### ICL Enzyme Assay and Inhibition Studies

*C. albicans* was grown for 24 hours in YNBL to induce expression of ICL. The cells were harvested by centrifugation at 10,000×*g* for 3 minutes. Cell-free extract was prepared as described previously [24], with some modifications. Briefly, the harvested cells were washed once with pre-chilled lysis buffer (100 mM potassium phosphate buffer, pH 7.5; 2 mM MgCl_2_; 1 mM DTT) and centrifuged again to collect the cell pellet. The cell pellet was re-suspended with the same lysis buffer and sonicated together with 0.7-mm glass beads for 3 minutes (30 seconds of bursting with 30 seconds cooling intervals). The cell lysate was centrifuged at 20,000×*g* at 4°C for 20 minutes to obtain the cell-free supernatant that was directly used in the enzyme assay.

The ICL enzyme assay was conducted in a 96-well plate formate and followed the protocol provided by Sigma-Aldrich Co., which was derived from [47], with some modifications. The 200 µL reaction volume consisted of 25 mM imidazole (pH 6.8), 5 mM MgCl_2_, 1 mM EDTA, 4 mM phenylhydrazine HCl, 1 mM DL-isocitric acid (substrate), and cell-free extract. The reaction was started right after the addition of substrate. Glyoxylate-phenylhydrazone formation was spectrophotometrically assessed at 324 nm using the Sunrise™ microplate absorbance reader after incubation at 30°C to determine the ICL activity in the reaction for the subsequent inhibition study. Similar reactions without substrate were prepared in parallel to serve as the blank. Potential ICL inhibitor (CAFF, ROS, and API) and all other plant reference compounds were further characterized for their inhibitory effects on ICL. The compounds were added to the reaction at final concentration of 50 µg/mL followed by incubation and measurement as previously described. A drug-free control was also prepared and the percent inhibition of ICL enzyme activity caused by each compound was calculated relative to the drug-free control.

### Minimum Inhibitory Concentration (MIC) Analysis of Identified Lead Compounds

The potential ICL inhibitors were subjected to MIC determination using the broth microdilution method according to the protocol described by EUCAST Definitive Document EDef 7.1 [46], with some modifications. Briefly, cells were cultivated in 96-well plates at a density of 0.5–2.5×10^5^ cfu/mL and separately treated at a gradient of concentrations from 64 to 0.125 mg/L (for fluconazole) and 1000–1.95 mg/L (for ITC and potential ICL inhibitors) in wells from column 1 to column 10, respectively. The drug-free control was included in the analysis for calculation of relative growth percentage. The cells in the 96-well plates were incubated and growth was measured as described previously. All MIC determinations were performed in triplicate. The MIC is defined as the lowest concentration that results in a growth reduction of ≥50% of that of the drug-free control.

### *in silico* Analysis of Drug-likeness and ADMET Properties

The potential ICL inhibitors identified in the experiments described above were analyzed for their ability to follow Lipinski's rule-of-five [26] by uploading them to the Molinspiration server (http://www.molinspiration.com/cgi-bin/properties) for calculation of their molecular properties. The chemical structures of the main compounds also were submitted to the admetSAR server (http://www.admetexp.org/predict/) for *in silico* prediction of their absorption, distribution, metabolism, excretion, and toxicity (ADMET) properties [25].

## Results

### The Alternative Screening Approach Identified Potential ICL Inhibitors

**Figure 3:**
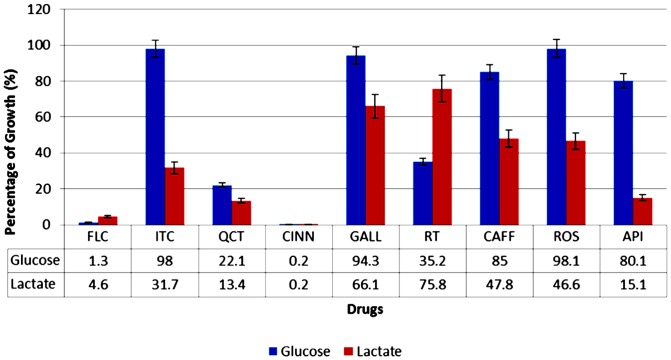
Alternative antifungal screening approach for *C. albicans* in YNB broth supplemented with glucose or lactate as the sole carbon source. The chart represents the averaged growth percentage with error bars of representation standard deviation. ITC is the known ICL inhibitor that served as the positive control in this experiment: it reduced the growth of *C. albicans* only in the lactate-supplemented medium. CAFF, ROS, and API caused growth reduction similar to that ITC, and hence were selected as potential ICL inhibitors for further analysis. QCT and CINN showed growth reduction in both media, which indicates non-specific inhibition or different targets. RT was the only compound that showed a glucose-specific pattern of growth reduction.

We utilized YNBL to screen for potential ICL inhibitors that reduce the growth of *C. albicans*. The cut off value was set at 40% growth reduction relative to the drug-free control. FLC and ITC were used as the positive controls. Primary screening yielded five potential compounds: QCT, CINN, CAFF, API, and ROS. Secondary screening, which included the assessment of growth in YNBG, further narrowed the list to three potential ICL inhibitors (CAFF, API, and ROS) that showed a lactate-specific pattern of growth reduction (Figure 3). Interestingly, RT from the secondary screening showed a glucose-specific pattern of growth reduction. These potential ICL inhibitors were further characterized for specific ICL-inhibition.

### The ICL Enzyme Assay Confirmed the Potential ICL Inhibitors

**Figure 4:**
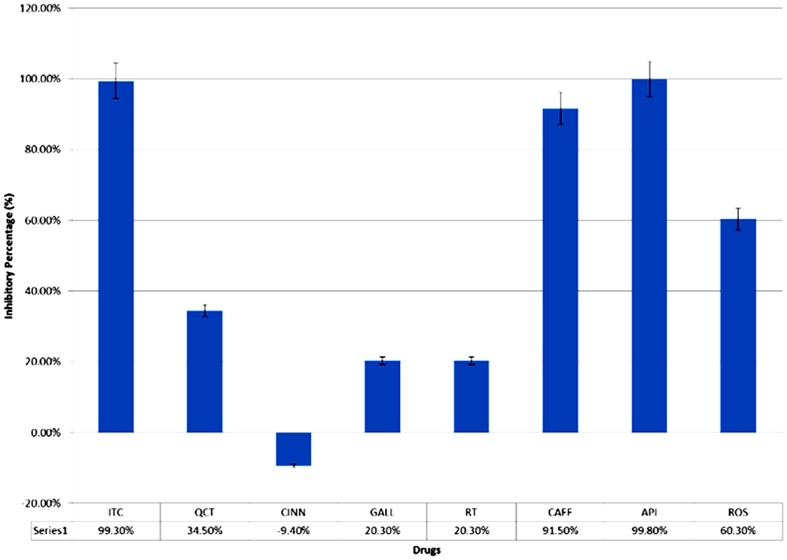
Inhibitory percentage of tested compounds in the ICL enzyme inhibition assay. The chart represents the averaged inhibitory percentage with error bars representing standard deviation. ITC is the known ICL inhibitor that served as the positive control in this experiment. CAFF, API, and ROS are the potential ICL inhibitors identified in the alternative screening experiment; they showed inhibition of ICL activity that was similar to that of ITC, with inhibitory percentage higher than 40% (in contrast to QCT, CINN, GALL, and RT).

Expression of ICL was induced in *C. albicans* to prepare cell-free extract for use in the ICL assay and inhibition studies. In the inhibition studies, the cutoff value was set at 40% inhibitory percentage, and ITC was included as the positive control. All of the known ICL inhibitor and potential inhibitors inhibited percentage values greater than 40% (ITC, 99.3%; CAFF, 91.5%; API, 99.8%; and ROS, 60.3%) in contrast to the non-lactate specific compounds (Figure 4).

### MIC Determination

The MICs for the tested compounds were determined in YNBL using the broth microdilution method. For FLC the MIC analysis was performed in both YNBL and YNBG. The results are summarized in Table 1. The MIC for FLC in YNBL (32 mg/L) was significantly higher than that in YNBG (0.5 mg/L), which suggests that carbon sources affect the on susceptibility of *C. albicans* to antifungal drugs. The MICs for the potential ICL inhibitors were much higher the 125 mg/L for API, 250 mg/L for ITC, 1000 mg/L for CAFÉ, and 1000 mg/L for ROS.

**Table 1:** MIC determination of potential inhibitors of ICL1 in *C. albicans* in YNB medium.

Potential ICL inhibitors/drugs	MIC (mg/L)
Fluconazole	32; 0.5 (YNBG)
Itaconic acid	250
Caffeic acid	1000
Rosmarinic acid	1000
Apigenin	125

### *in silico* Analysis of Drug-likeness and ADMET Properties of Potential ICL Inhibitors

The molecular structures of FLC, ITC, CAFF, ROS, and API were submitted to the Molinspiration and admetSAR servers to determine their drug-likenesses and ADMET properties. All of the tested compounds were roughly compliant with Lipinski's rule-of-five [26] without any violation with respect to lipophilicity (miLogP), molecular weight, number of hydrogen bond donors, and number of hydrogen bond acceptors (Table 2). The bioavailability and toxicity assessments of the compounds were conducted based on their ADMET properties (Table 3). FLC, ITC, CAFF, and ROS had no significant ADMET properties that would cause adverse effects in humans, whereas API was predicted to be a potential inhibitor of CYP 1A2, 2C9, 2C19, and 3A4, with high CYP inhibitory promiscuity. None of the submitted compounds were identified as potential inhibitors of the human ether-a-go-go-related gene (HERG) or were positive for AMES toxicity or carcinogenicity.

**Table 2:** Lipinski's rule-of-five drug-likeness properties of potential ICL inhibitors.

Compound	FLC	ITC	CAFF	ROS	API
**Mi LogP**	−0.118	−0.343	0.941	1.626	2.463
**TPSA**	81.664	74.598	77.755	144.516	90.895
**n atoms**	22	9	13	26	20
**MW**	306.276	130.099	180.159	360.318	270.24
**n ON**	7	4	4	8	5
**n OHNH**	1	2	3	5	3
**n violations**	0	0	0	0	0
**n rotb**	5	3	2	7	1
**MV**	248.957	111.171	154.497	303.539	224.049

Abbreviations: Mi LogP (hydrophobicity measurement: octanol/water partition coefficient); TPSA (topological polar surface area); n atoms (number of atoms); MW (molecular weight); n ON (hydrogen bond acceptor); n OHNH (number of hydrogen bond donor); n violations (number of Lipinski's rule-of-five violations); n rotb (number of rotatable bonds); MV (molecular volume).

High bioavailability are more probable for a compound when there are ≤5 hydrogen bond donors, ≤10 hydrogen bond acceptors, molecular weight ≤500, and Mi LogP ≤5; violation more than one of these rules may have problem with bioavailability [26].

**Table 3:** ADMET properties of potential ICL inhibitors.

ADMET	FLC	ITC	CAFF	API	ROS
**BBB**	+	+	-	+	+
**HIA**	+	+	+	+	+
**Caco-2**	+	-	+	+	-
**Aqueous solubility**	– 1.8626	–0.7363	–1.6939	–2.7765	–3.2050
**P-gp**					
Substrate	-	-	-	-	+
Inhibitor	-	-	-	-	-
**CYP450 substrate**					
CYP450 2C9	-	-	-	-	-
CYP450 2D6	-	-	-	-	-
CYP450 3A4	-	-	-	-	-
**CYP450 inhibitor**					
CYP450 1A2	-	-	-	+	-
CYP450 2C9	-	-	-	+	-
CYP450 2C19	_+_	-	-	+	-
CYP450 2D6	-	-	-	-	-
CYP450 3A4	-	-	-	+	-
**CYP IP**	low	low	low	low	low
**ROCT**	-	-	-	-	-
**HERG inhibition**					
HERG-I	weak	weak	weak	weak	weak
HERG-II	-	-	-	-	-
**AMES toxicity**	-	-	-	-	-
**Carcinogen**	-	-	-	-	-
**RAT, LD_50_ mol/kg**	2.4136	2.4525	1.4041	2.6983	2.3234
**FT, pLC_50_ mg/L**	high, 1.4529	high, 0.8180	high, 0.7921	high, 0.8038	high, –0.1231
**TPT, pIGL50 µg/L**	high, 0.5995	low, −0.5487	high, −0.2480	high, 0.3341	high, 0.8320

Abbreviations: BBB (blood-brain barrier); HIA (human intestinal absorption); Caco-2 (Caco-2 permeability); P-gp (P-glycoprotein); ROCT (renal organic cation transporter); CYP450 (cytochrome P450); CYP IP (CYP inhibitory promiscuity); HERG (human ether-a-go-go-related genes); RAT (rat acute toxicity); FT (fish toxicity); TPT (*Tetrahymena pyriformis* toxicity).

BBB, HIA, Caco-2, and aqueous solubility indicate the bioavailability of the drug in terms of absorption into the human body; P-gp, CYP450, and CYP IP indicate the metabolism, clearance, and risk of drug-drug interaction with the co-administered drug; ROCT indicates the renal excretion of the drug; HERG indicates the risk of cardiotoxicity caused by the drug; AMES toxicity indicates the risk of carcinogenicity and genotoxicity caused by the drug; RAT, LD_50_ is the lethal dosage of drug when tested on mice; FT and TPT are the environmental risk assessments of the drug based on fish and *Tetrahymena pyriformis* as environmental indicators, respectively.

## Discussion

ICL is one of the key enzymes of the glyoxylate cycle, which enables *C. albicans* to survive in a wide variety of glucose-depleted niches and establish candidiasis [10], [12], [13], [14]. Recently, ICL was found to be essential for virulence of several other pathogens, and it has been explored as a potential drug target in some of them [18], [19], [20], [21], [22]. Furthermore, because there is no known human ortholog of this enzyme, it an attractive antifungal target for drug discovery to treat candidiasis with minimal adverse side effects and toxicity. In this study, we screened and identified potential ICL inhibitors from a collection of selected plant reference compounds (Figure 2) as potential antifungal drugs to treat candidiasis. These selected plant reference compounds are phenolic or polyphenolic compounds (flavonoids), which are among the main secondary metabolites in plants, and an integral part of the human diet [27]. Screening of these plant-derived phenolic and polyphenolic compounds for antifungal drugs is more promising approach as they known to have antioxidant, anti-inflammatory, and antimicrobial properties [28], [29].

Traditional antifungal screening methods mostly utilize a medium supplemented with glucose as the carbon source [30], [31], [45], [46]. However, this approach may not be relevant to the host niches and therefore might undervalue the potential of certain target-specific compounds. Considering the importance of the target enzyme for *C. albicans* (ICL1), we utilized a minimal defined medium (YNB) supplemented with lactate in place of glucose to mimic the environment that *C. albicans* encounters in host niches. We used lactate as an alternative carbon source because of its physiological relevance. Lactate is found in ingested foods and is generated by bacteria in the gastrointestinal and urogenital tracts [32]. Lactate is also a component of solutions used in surgery or to treat burn injuries (e.g., lactated Ringer's solutions and Hartmann's solution) [1]. Moreover, the activation of lactate transporters in *C. albicans* cells upon being engulfed by macrophages also suggests that a high concentration of lactate is present in macrophages [16]. In this study, the alternative screening approach identified three potential ICL inhibitors (CAFF, ROS, and API), all of which showed a lactate-specific pattern of growth reduction. The secondary screening consisted of triplicate screening in both YNBL and YNBG; the latter was included to identify non-specific compounds that targeted cell activity not associated with the glyoxylate cycle, which were QCT and CINN in this study. The secondary screening also served to eliminate any non-specific, detergent-like compounds that caused growth reduction of *C. albicans* due to cell lysis or general toxicity rather than a defined mechanism of action. From this secondary screening, we identify three CAFF, ROS, and API as showing a lactate-specific pattern of growth inhibition. Interestingly, RT shows glucose-specific pattern of growth reduction in the secondary screening, which suggests that it might target the cellular pathway associated with glucose assimilation.

To confirm that the growth reductions observed using the alternative screening approach were associated with specific inhibition of ICL, the compounds were screened for inhibition of ICL activity. The tested compounds inhibited inhibition percentages of more than 40%, with the greatest inhibition by API (99.8%), followed by ITC (99.3%), CAFF (91.5%), and ROS (60.3%). The inhibitory property of these compounds is not conclusive, as they might inhibit ICL activity in combination with other cellular components from the crude extract. However, because the regulation of ICL activity in yeast occurs at the posttranslational level [24], further purification of the ICL enzyme was not conducted because we wanted to prevent degradation or inactivation of the enzyme. These potential ICL inhibitors were also subjected to MIC determination for *C. albicans* in YNBL. The MIC was lowest for API (125 mg/L), followed by ITC (250 mg/L), CAFF (1000 /L), and ROS (1000 mg/L). Although the high MICs values especially for CAFF and ROS, suggest that growth inhibition might be due to the general toxicity effects of overdoses, the alternative screening results rule out by this possibility, (i.e., growth percentage higher than 80% for *C. albicans* treated with 1000 /L of CAFF, ROS, and API in YNBG) (Figure 3). Interestingly, the MIC of FLC in YNBL was higher than that in YNBG. This difference in MIC when cells were grown with different carbon sources was not surprising, as lactate-grown *C. albicans* cells were previously reported to exhibit increased resistance to certain antifungal drugs, probably because the carbon source strongly influences cell wall properties, virulence, and susceptibility to antifungal drugs [8].

In drug discovery, only a small proportion of promising compounds reach the market due to a high failure rate at the clinical trial stage, mostly due to toxicity, adverse side effects on recipients and poor ADMET properties [25]. Knowledge about ADMET properties is especially useful when conducting drug safety assessments and it can be very important for reducing the failure rate at the clinical stage [33]. ADMET properties can be assessed via experimentation, via *in silico* analysis [25], or as in this case, the toxicity profiles of the potential ICL inhibitors can be retrieved from previous reports because they have been studied as drugs for use in other medical situations. Experimental assessment of ADMET properties is expensive, labor intensive, and cannot meet the demands of high throughput drug screening. However, *in silico* techniques can predict ADMET properties based on the structure-activity relationship of a given compound [25]. In this study, we used this alternative approach by submitting the compound structures to the Molinspiration and admetSAR servers for *in silico* analysis. The Molinspiration server uses Lipinski's rule-of-five to analyze the drug-likeness of a given compound [26]. In this study, none of the tested compounds violated any of the rules (Table 2). Although Lipinski's rule-of-five is useful in early drug assessment, it is too simplistic and the ADMET properties of a drug administered into a recipient's body are important to know at later stages of assessment process.

The analysis performed on the admetSAR server revealed that of the potential ICL inhibitors tested only API potentially would cause adverse side effects in recipients. API exhibits high CYP inhibitory promiscuity, as it inhibited most of the cytochrome P450 isoforms, including CYP450 1A2, 2C9, 2C19, and 3A4. The cytochrome P450 superfamily plays an important role in drug metabolism and clearance in the liver, and the most important isoforms are CYP1A2, CYP2A6, CYP2C9, CYP2C19, CYP2D6, CYP2E1, and CYP3A4 [34]. Thus, inhibition of cytochrome P450 isoforms might cause drug-drug interactions in which co-administered drugs (substrates for the inhibited P450 isoforms) fail to be metabolized and accumulate to toxic levels [35]. The analysis also showed ROS to be a substrate for P-glycoprotein, which effluxes drugs and various compounds so that they can be further metabolized for clearance [36]. If P-glycoprotein is induced, drugs in the medication would be transported out of the cells at a greater rate and could lead to therapeutic failure because the drug concentration would be lower than expected [36]. Therefore, dosage control and knowledge of co-administered drugs must be considered to reduce therapeutic failure.

Although the ADMET analysis indicated that none of the potential ICL inhibitors exhibited any significant potential for toxicity to humans (Table 3), previously published toxicity profiles of CAFF and API suggest otherwise. API is a dietary flavonoid reported to have medicinal values for treating different diseases [37], but it was shown to cause oxidative stress-induced liver damage at higher doses in Swiss mice [38]. Another study demonstrated that API triggers apoptosis in red blood cells by stimulation of Ca^2+^ influx, ceramide formation, and ATP depletion in cells [39]. CAFF has been identified as an active antioxidant and inhibitor of carcinogenesis [40], but studies of CAFF toxicity have had mixed results. Some studies suggest that it inhibits carcinogenesis, whereas other experiments show carcinogenic effects in experimental mice [41]. ROS is a caffeic acid ester commonly found in plants that acts as a defense compound [42]. Previous studies demonstrated that ROS has a number of biological activities. ROS has been found to exert anti-oxidation and anti-inflammatory effects that may be useful in reducing cardiovascular diseases and in the treatment of acute lung injury, respectively [43], [44]. De Oliveira [44] reported that ROS could protect against ethanol-induced DNA damage in mice. However, studies of ROS toxicity are scarce in the literature.

## Conclusions

This study was conducted to identify potential antifungal drugs effective against *C. albicans* by targeting the ICL enzyme of the glyoxylate cycle using an alternative screening approach in minimal defined medium supplemented with lactate as the single carbon source. We identified three potential ICL inhibitors (CAFF, API, and ROS) and explored them in further detail to better understand their activity against *C. albicans* and their potential to cause adverse side effects in recipients. While there is still much to be learned before these compounds can be considered viable drug candidates, this study indicates that new compounds can be identified from the existing collection of reference compounds and that new pathways can be specifically targeted via alternative target-based screening approaches.

## References

[1] Pfaller MA, Diekema DJ (2010) Epidemiology of Invasive Mycoses in North America. Currr Rev Microbiol 36: 1–53 10.3109/10408410903241444 20088682

[2] Pappas PG, Kauffman CA, Andes D, Benjamin DK Jr, Calandra TF, et al (2009) Clinical practice guidelines for the management of candidiasis: 2009 update by the Infectious Diseases Society of America. Clin Infect Dis 48: 503–535 10.1086/596757 19191635 PMC7294538

[3] Perlroth J, Choi B, Spellberg B (2007) Nosocomial fungal infections: epidemiology, diagnosis, and treatment. Med Mycol 45: 321–346 10.1080/13693780701218689 17510856

[4] Mayer FL, Wilson D, Hube B (2013) *Candida albicans* pathogenicity mechanisms. Virulence 4: 119–128 10.4161/viru.22913 23302789 PMC3654610

[5] Fleck CB, Schöbel F, Brock M (2011) Nutrient acquisition by pathogenic fungi: nutrient availability, pathway regulation, and differences in substrate utilization. Int J Med Microbiol 301: 400–407 10.1016/j.ijmm.2011.04.007 21550848

[6] Chandrasekar P (2011) Management of invasive fungal infections: a role for polyenes. J Antimicrob Chemother 66: 457–465 10.1093/jac/dkq479 21172787

[7] Brock M (2009) Fungal metabolism in host niches. Curr Opin Microbiol 12: 371–376 10.1016/j.mib.2009.05.004 19535285

[8] Ene IV, Adya AK, Wehmeier S, Brand AC, MacCallum DM, et al (2012) Host carbon sources modulate cell wall architecture, drug resistance and virulence in a fungal pathogen. Cell Microbiol 14: 1319–1335 10.1111/j.1462-5822.2012.01813.x 22587014 PMC3465787

[9] Lorenz MC, Fink GR (2002) Life and death in a macrophage: role of the glyoxylate cycle in virulence. Eukaryotic Cell 1: 657–662. 12455685 10.1128/EC.1.5.657-662.2002 PMC126751

[10] Dunn MF, Ramírez-Trujillo JA, Hernández-Lucas I (2009) Major roles of isocitrate lyase and malate synthase in bacterial and fungal pathogenesis. Microbiol (Reading, Engl) 155: 3166–3175 10.1099/mic.0.030858-0 19684068

[11] Kondrashov FA, Koonin EV, Morgunov IG, Finogenova TV, Kondrashova MN (2006) Evolution of glyoxylate cycle enzymes in Metazoa: evidence of multiple horizontal transfer events and pseudogene formation. Biol Direct 1: 31 10.1186/1745-6150-1-31 17059607 PMC1630690

[12] Fernández-Arenas E, Cabezón V, Bermejo C, Arroyo J, Nombela C, et al (2007) Integrated proteomics and genomics strategies bring new insight into *Candida albicans* response upon macrophage interaction. Mol Cell Proteomics 6: 460–478 10.1074/mcp.M600210-MCP200 17164403

[13] Marcil A, Gadoury C, Ash J, Zhang J, Nantel A, et al (2008) Analysis of PRA1 and its relationship to *Candida albicans*- macrophage interactions. Infect Immun 76: 4345–4358 10.1128/IAI.00588-07 18625733 PMC2519410

[14] Lorenz MC, Fink GR (2001) The glyoxylate cycle is required for fungal virulence. Nature 412: 83–86 10.1038/35083594 11452311

[15] Prigneau O, Porta A, Poudrier JA, Colonna-Romano S, Noël T, et al (2003) Genes involved in beta-oxidation, energy metabolism and glyoxylate cycle are induced by *Candida albicans* during macrophage infection. Yeast 20: 723–730 10.1002/yea.998 12794933

[16] Lorenz MC, Bender JA, Fink GR (2004) Transcriptional Response of *Candida albicans* upon Internalization by Macrophages. Eukaryotic Cell 3: 1076–1087 10.1128/EC.3.5.1076-1087.2004 15470236 PMC522606

[17] Fradin C, Kretschmar M, Nichterlein T, Gaillardin C, d' Enfert C, et al (2003) Stage-specific gene expression of *Candida albicans* in human blood. Mol Microbiol 47: 1523–1543. 12622810 10.1046/j.1365-2958.2003.03396.x

[18] Ebel F, Schwienbacher M, Beyer J, Heesemann J, Brakhage AA, et al (2006) Analysis of the regulation, expression, and localisation of the isocitrate lyase from *Aspergillus fumigatus*, a potential target for antifungal drug development. Fungal Genet Biol 43: 476–489 10.1016/j.fgb.2006.01.015 16603391

[19] Gengenbacher M, Rao SPS, Pethe K, Dick T (2010) Nutrient-starved, non-replicating *Mycobacterium tuberculosis* requires respiration, ATP synthase and isocitrate lyase for maintenance of ATP homeostasis and viability. Microbiol (Reading, Engl) 156: 81–87 10.1099/mic.0.033084-0 19797356

[20] Marrero J, Rhee KY, Schnappinger D, Pethe K, Ehrt S (2010) Gluconeogenic carbon flow of tricarboxylic acid cycle intermediates is critical for *Mycobacterium tuberculosis* to establish and maintain infection. PNAS 107: 9819–9824 10.1073/pnas.1000715107 20439709 PMC2906907

[21] Krátký M, Vinšová J (2012) Advances in mycobacterial isocitrate lyase targeting and inhibitors. Curr Med Chem 19: 6126–6137. 23092127 10.2174/092986712804485782

[22] Van Schaik EJ, Tom M, Woods DE (2009) *Burkholderia pseudomallei* isocitrate lyase is a persistence factor in pulmonary melioidosis: implications for the development of isocitrate lyase inhibitors as novel antimicrobials. Infect Immun 77: 4275–4283 10.1128/IAI.00609-09 19620343 PMC2747945

[23] Van Acker H, Sass A, Bazzini S, De Roy K, Udine C, et al (2013) Biofilm-grown *Burkholderia cepacia* complex cells survive antibiotic treatment by avoiding production of reactive oxygen species. PLoS ONE 8: e58943 10.1371/journal.pone.0058943 23516582 PMC3596321

[24] Cruz AH da S, Brock M, Zambuzzi-Carvalho PF, Santos-Silva LK, Troian RF, et al (2011) Phosphorylation is the major mechanism regulating isocitrate lyase activity in *Paracoccidioides brasiliensis* yeast cells. FEBS J 278: 2318–2332 10.1111/j.1742-4658.2011.08150.x 21535474

[25] Cheng F, Li W, Zhou Y, Shen J, Wu Z, et al (2012) admetSAR: a comprehensive source and free tool for assessment of chemical ADMET properties. J Chem Inf Model 52: 3099–3105 10.1021/ci300367a 23092397

[26] Lipinski CA (2004) Lead- and drug-like compounds: the rule-of-five revolution. Drug Discov Today: Technol 1: 337–341 10.1016/j.ddtec.2004.11.007 24981612

[27] Gautam MK, Gangwar M, Nath G, Rao CV, Goel RK (2012) In–vitro antibacterial activity on human pathogens and total phenolic, flavonoid contents of *Murraya paniculata* Linn. leaves. Asian Pac J Trop Biomed 2: S1660–S1663 10.1016/S2221-1691(12)60472-9

[28] Klančnik A, Piskernik S, Jeršek B, Možina SS (2010) Evaluation of diffusion and dilution methods to determine the antibacterial activity of plant extracts. J Microbiol Methods 81: 121–126 10.1016/j.mimet.2010.02.004 20171250

[29] Askun T, Tekwu EM, Satil F, Modanlioglu S, Aydeniz H (2013) Preliminary antimycobacterial study on selected Turkish plants (Lamiaceae) against *Mycobacterium tuberculosis* and search for some phenolic constituents. BMC Complem Altern Med 13: 365 10.1186/1472-6882-13-365 PMC3878028 24359458

[30] Krysan DJ, Didone L (2008) A high-throughput screening assay for small molecules that disrupt yeast cell integrity. J Biomol Screen 13: 657–664 10.1177/1087057108320713 18626115

[31] Kitamura A, Someya K, Hata M, Nakajima R, Takemura M (2009) Discovery of a Small-Molecule Inhibitor of β-1,6-Glucan Synthesis. Antimicrob Agents Chemother 53: 670–677 10.1128/AAC.00844-08 19015325 PMC2630612

[32] Ene IV, Cheng S-C, Netea MG, Brown AJP (2013) Growth of *Candida albicans* cells on the physiologically relevant carbon source lactate affects their recognition and phagocytosis by immune cells. Infect Immun 81: 238–248 10.1128/IAI.01092-12 23115042 PMC3536122

[33] Merlot C (2010) Computational toxicology—a tool for early safety evaluation. Drug Discov Today 15: 16–22 10.1016/j.drudis.2009.09.010 19835978

[34] Vasanthanathan P, Taboureau O, Oostenbrink C, Vermeulen NPE, Olsen L, et al (2009) Classification of cytochrome P450 1A2 inhibitors and noninhibitors by machine learning techniques. Drug Metab Dispos 37: 658–664 10.1124/dmd.108.023507 19056915

[35] Lynch T, Price A (2007) The effect of cytochrome P450 metabolism on drug response, interactions, and adverse effects. Am Fam Physician 76: 391–396. 17708140

[36] Levin GM (2012) P-glycoprotein: why this drug transporter may be clinically important. Curr Psychiatry 11: 38–40.

[37] Chuang C-M, Monie A, Wu A, Hung C-F (2009) Combination of apigenin treatment with therapeutic HPV DNA vaccination generates enhanced therapeutic antitumor effects. J Biomed Sc 16: 49 10.1186/1423-0127-16-49 19473507 PMC2705346

[38] Singh P, Mishra SK, Noel S, Sharma S, Rath SK (2012) Acute Exposure of Apigenin Induces Hepatotoxicity in Swiss Mice. PLoS ONE 7: e31964 10.1371/journal.pone.0031964 22359648 PMC3281105

[39] Zbidah M, Lupescu A, Jilani K, Fajol A, Michael D, et al (2012) Apigenin-induced suicidal erythrocyte death. J Agric Food Chem 60: 533–538 10.1021/jf204107f 22132906

[40] Gülçin İ (2006) Antioxidant activity of caffeic acid (3,4-dihydroxycinnamic acid). Toxicol 217: 213–220 10.1016/j.tox.2005.09.011 16243424

[41] Hirose M, Takesada Y, Tanaka H, Tamano S, Kato T, et al (1998) Carcinogenicity of antioxidants BHA, caffeic acid, sesamol, 4-methoxyphenol and catechol at low doses, either alone or in combination, and modulation of their effects in a rat medium-term multi-organ carcinogenesis model. Carcinogenesis 19: 207–212. 9472713 10.1093/carcin/19.1.207

[42] Petersen M, Simmonds MSJ (2003) Rosmarinic acid. Phytochem 62: 121–125. 10.1016/s0031-9422(02)00513-7 12482446

[43] Karthik D, Viswanathan P, Anuradha CV (2011) Administration of rosmarinic acid reduces cardiopathology and blood pressure through inhibition of p22phox NADPH oxidase in fructose-fed hypertensive rats. J Cardiovasc Pharmacol 58: 514–521 10.1097/FJC.0b013e31822c265d 21795992

[44] De Oliveira NCD, Sarmento MS, Nunes EA, Porto CM, Rosa DP, et al (2012) Rosmarinic acid as a protective agent against genotoxicity of ethanol in mice. Food Chem Toxicol 50: 1208–1214 10.1016/j.fct.2012.01.028 22306517

[45] CLSI (2008) Reference method for broth dilution antifungal susceptibility testing of yeasts, 3rd informational supplement. M27–S3. Clinical and Laboratory Standards Institute, Wayne, PA.

[46] Subcommittee on Antifungal Susceptibility Testing (AFST) of the ESCMID European Committee for Antimicrobial Susceptibility Testing (EUCAST) (2008) EUCAST definitive document EDef 7.1: method for the determination of broth dilution MICs of antifungal agents for fermentative yeasts. Clin Microbiol Infect 14: 398–405 10.1111/j.1469-0691.2007.01935.x 18190574

[47] Chell RM, Sundaram TK, Wilkinson AE (1978) Isolation and characterization of isocitrate lyase from a thermophilic *Bacillus* sp. Biochem J 173: 165–177. 687365 10.1042/bj1730165 PMC1185759

